# Population Dynamics and Evolutionary History of the Weedy Vine *Ipomoea hederacea* in North America

**DOI:** 10.1534/g3.114.011700

**Published:** 2014-06-03

**Authors:** Brandon E. Campitelli,, John. R. Stinchcombe

**Affiliations:** * Department of Integrative Biology, College of Natural Sciences, University of Texas at Austin, Austin, Texas 78712; † Department of Ecology and Evolutionary Biology, University of Toronto, Toronto, ON, Canada, M5S 3B2; ‡ Centre for the Analysis of Genome Evolution and Function, University of Toronto, Toronto ON, Canada, M5S 3B2

**Keywords:** population expansion, structure, bottlenecks, metapopulation, leaf shape

## Abstract

Disentangling the historical evolutionary processes that contribute to patterns of phenotypic and genetic variation is important for understanding contemporary patterns of both traits of interest and genetic diversity of a species. *Ipomoea hederacea* is a self-compatible species whose geographic origin is contested, and previous work suggests that although there are signals of adaptation (significant leaf shape and flowering time clines), no population structure or neutral genetic differentiation of *I. hederacea* populations was detected. Here, we use DNA sequence data to characterize patterns of genetic variation to establish a more detailed understanding of the current and historical processes that may have generated the patterns of genetic variation in this species. We resequenced ca. 5000 bp across 7 genes for 192 individuals taken from 24 populations in North America. Our results indicate that North American *I. hederacea* populations are ubiquitously genetically depauperate, and patterns of nucleotide diversity are consistent with population expansion. Contrary to previous findings, we discovered significant population subdivision and isolation-by-distance, but genetic structure was spatially discontinuous, potentially implicating long-distance dispersal. We further found significant genetic differentiation at sequenced loci but nearly fourfold stronger differentiation at the leaf shape locus, strengthening evidence that the leaf shape locus is under divergent selection. We propose that North American *I. hederacea* has experienced a recent founder event, and/or population dynamics are best described by a metapopulation model (high turnover and dispersal), leading to low genetic diversity and a patchy genetic distribution.

Understanding the interplay between demographic and adaptive processes is important for making biologic interpretations about patterns of both phenotypic and genetic variation across contemporary populations (Eckhart *et al.* 2011; Moeller *et al.* 2011; Domingues *et al.* 2012). Determining the relative importance of these processes has been a central goal in evolutionary biology; however, distinguishing between demographic and adaptive hypotheses is often a challenge because of major historical events (*e.g.*, bottlenecks) and because current population dynamics may not reflect the past conditions that shaped patterns of genetic variation, geographic distributions, population structure, and adaptation. For example, the advance and subsequent retreat of glaciers ~20,000 years ago has played a major role in shaping the geographical distributions and patterns of genetic variation of many plants and animals in North America (NA; reviewed by Soltis *et al.* 2006). Analyses of neutral population genetic variation offers a window to these processes, at least with respect to the patterns imprinted in the genome, and here we apply this approach to a weedy annual plant, *Ipomoea hederacea*, to better understand its population history in North America.

One approach to disentangling issues of population history is to explore neutral molecular variation in a species, where signatures of historical demography and adaptation may be stored. Data on genetic variation can then be placed into the well-established framework of theoretical population genetics, such that inferences about population history can be made (Charlesworth *et al.* 2003). For example, nonrandom geographical patterns in molecular diversity can provide key insights about the genetic structure of populations (Charlesworth *et al.* 2003), the center(s) of colonization or invasion (*e.g.*, Ness *et al.* 2010), and provide the routes along which range expansion is occurring (Soltis *et al.* 2006), all of which can be used to determine whether demography or adaptation—or a combination of both—dominate the evolutionary history of a species. Both demographic events and stochastic evolutionary processes are expected to influence all genetic variants across the genome, whereas natural selection will have locus-specific effects (Cavalli-Sforza 1966; Lewontin and Krakauer 1973; Berry and Kreitman 1993; Vasemägi 2006). Hence, focal loci under selection may display patterns that differ markedly from the remaining neutral genetic background.

Ivyleaf morning glory, *Ipomoea hederacea*, possesses an interesting mix of patterns for both ecologically important traits and neutral variation, suggesting population genetic knowledge would be useful for reconciling contrasting patterns and for interpreting its evolutionary history. First, although its current range is well-documented (Bright 1998), its origin is uncertain; several have claimed that it is an invasive species in the eastern United States that originated from tropical America (Strausbaugh and Core 1964; Long and Lakela 1971; Wunderlin 1982), whereas others declare that it is native to the southeastern United States (Mohr 1901), and early floras place it in Eastern NA from the early 1800s (Michaux 1805; Pursh 1814). Second, despite its unclear native status in NA, it exhibits clinal patterns for several traits that are beyond neutral expectations, implicating natural selection; leaf shape (Bright 1998; Campitelli and Stinchcombe 2013a), flowering phenology (Klingaman and Oliver 1996; Stock *et al.* 2014), growth rate, and several aspects of floral morphology (Stock *et al.* 2014) all exhibit significant latitudinal clines. Third, previous work, using 173 AFLP loci suggests that *I. hederacea* populations in NA are not genetically structured or differentiated, and they do not exhibit isolation-by-distance (IBD; Campitelli and Stinchcombe 2013a), which was surprising given past evidence for high selfing rates (*SR*s; Ennos 1981; Hull-Sanders *et al.* 2005), and so genetic differences between populations are expected to have accumulated over time. Therefore, this species is a useful test case for characterizing patterns of polymorphism and diversity across the genome and as a point of comparison for understanding its origin, population genetic structure, adaptation, and the demographic processes governing its evolutionary history in NA.

In the current study we use Sanger sequencing to collect DNA sequence data for a large sample of individuals from many populations in *I. hederacea*’s eastern NA range and apply basic molecular population genetic tools to characterize patterns of polymorphism, diversity, and population structure. Specifically, we sought to address the following three questions regarding the evolutionary history of *I. hederacea* in NA: (1) Is there evidence of hierarchical population structure and genetic differentiation at the nucleotide level? (2) How genetically diverse are *I. hederacea* populations, and are there geographical patterns associated with nucleotide diversity? (3) What are the potential historical processes that have generated the patterns that we observe now?

## Materials and Methods

### Natural history

**Figure 1:**
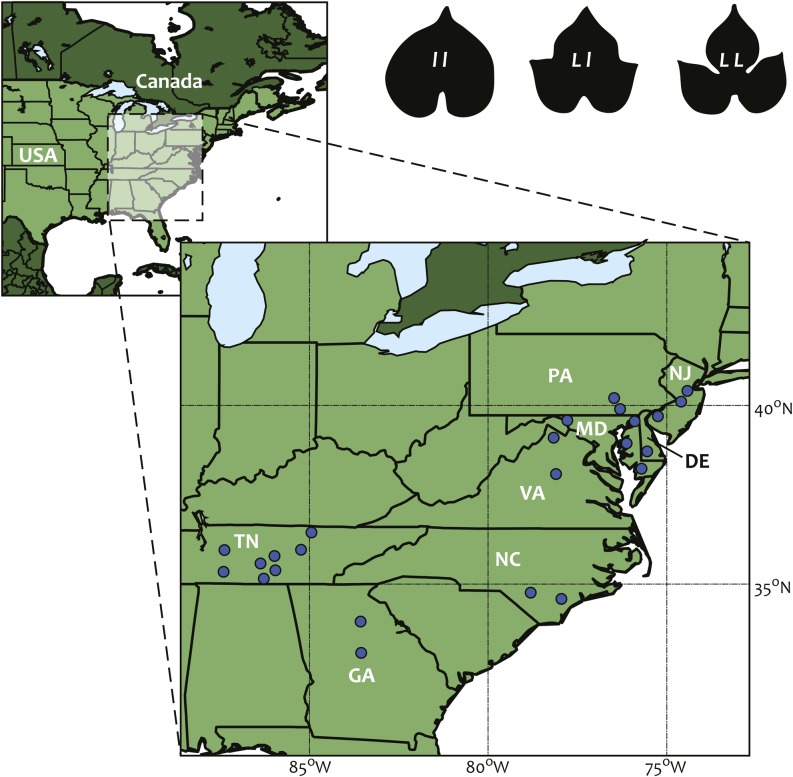
Map showing location of each *I. hederacea* population, and an example of the three distinct leaf shape genotypes; entire-shaped (*ll*), heterozygotes (*Ll*), and lobed (*LL*).

*Ipomoea hederacea* (L.) Jaquin (Convolvulacaea) is an herbaceous weedy vine that has a NA range spanning from the southern United States (*e.g.*, Florida to Texas) up through the Great Lakes region (Figure 1). It typically inhabits disturbed soils in open habitats such as the edges of agricultural fields and roadside ditches. Its annual lifecycle begins in late spring (May to June) and continues until a season ending frost. It initiates flowering approximately 8 wk after germination and produces flowers continuously until senescence. Its hermaphroditic flowers mainly self-fertilize, with a wide range of *SR*s (Ennos 1981: 93%; Hull-Sanders *et al.* 2005: 19–93%, $\bar{x}=63\%$), estimated depending on location, year measured, marker types, and methods. Each flower yields a single fruit that rapidly matures, and seeds are primarily dispersed through gravity, although rare long-distance dispersal may occur (Campitelli and Stinchcombe 2013a), most likely from agricultural activity, as in other *Ipomoea* species (Epperson and Clegg 1986).

### Population sampling and leaf shape determination

In previous work (Campitelli and Stinchcombe 2013a), we sampled seeds from 77 populations spanning the full leaf shape clinal range of *I. hederacea*. Here, we us a subset of 24 of these populations, which we selected based on the following criteria; we chose 12 populations *north and south* of the putative clinal boundary determined in Campitelli and Stinchcombe (2013a), and we used populations with at least 8 individuals from each for a total of 192 maternal lines. Seeds were originally collected in the field from plants that were separated by at least 2 m to avoid collecting from immediate siblings. Population locations are shown in Figure 1, and summary statistics for populations are presented in Table 1. We characterized leaf shape genotypes for each line by using the criteria and experimental procedures described by Campitelli and Stinchcombe (2013a); in summary, we planted 10 field-collected seeds from each maternal line in a greenhouse and applied the rules of Mendelian inheritance to assign the most likely genotype to each maternal parent based on leaf shape segregation within its 10 progeny.

**Table 1:** Summary of leaf shape statistics and diversity indices of 24 North American populations of *Ipomoea hederacea*

Population	% L Allele*^a^*	% l Allele*^a^*	θ_W_*^b^*	θ_π_*^c^*	Taj D*^d^*	Sim D*^e^*	% Low*^f^*	% High*^f^*
GA80	0.25	0.75	0.00105	0.00112	0.12	−0.03	59.90	40.20
GA78	0.63	0.37	0.00088	0.00102	−0.17	−0.03	42.10	57.90
NC35	0.00	1.00	0.00071	0.00043	−1.00	−0.02	9.80	90.20
NC42	0.00	1.00	0.00088	0.00101	0.17	−0.03	62.80	37.20
TN61	0.13	0.87	0.00126	0.00110	−0.32	−0.03	31.30	68.70
TN55	0.31	0.69	0.00162	0.00118	−0.74	−0.02	8.80	91.20
TN62	0.50	0.50	0.00087	0.00081	−0.54	−0.03	19.10	80.90
TN57	0.25	0.75	0.00098	0.00108	0.01	−0.05	54.60	45.40
TN65	0.56	0.44	0.00070	0.00069	−0.33	−0.04	34.30	65.70
TN64	0.19	0.81	0.00070	0.00069	−0.45	−0.02	30.10	69.90
TN63	0.37	0.63	0.00175	0.00131	−0.43	−0.03	22.40	77.60
TN59	0.13	0.87	0.00080	0.00084	−0.34	−0.10	36.30	63.70
DE19	1.00	0.00	0.00126	0.00125	−0.28	−0.20	45.00	55.00
MD22	1.00	0.00	0.00144	0.00153	0.28	−0.11	79.60	20.40
MD23	1.00	0.00	0.00108	0.00114	0.04	−0.02	54.30	45.70
VA06	0.00	1.00	0.00174	0.00123	−0.53	−0.02	17.40	82.60
VA10	1.00	0.00	0.00089	0.00080	−0.02	−0.03	51.90	48.10
MD17	1.00	0.00	0.00135	0.00135	−0.12	−0.02	44.20	55.90
MD11	1.00	0.00	0.00089	0.00095	−0.11	−0.01	44.50	55.50
NJ32	1.00	0.00	0.00071	0.00077	−0.25	−0.03	37.00	63.00
PA16	0.25	0.75	0.00123	0.00115	0.03	−0.02	53.20	46.80
NJ30	1.00	0.00	0.00091	0.00124	1.14	−0.01	93.20	6.80
PA15	1.00	0.00	0.00071	0.00071	−0.38	−0.03	29.90	70.10
NJ31	0.25	0.75	0.00053	0.00071	0.43	−0.04	72.50	27.50
Average	0.53	0.47	0.00324	0.00114	−0.84	−0.02	1.09	98.91

Populations are listed from most southern to most northern.

a Frequencies of the lobing (*L*) and entire (*l*) leaf shape alleles.

b Number of silent segregating polymorphisms per site.

c Average number of silent pairwise nucleotide differences per site.

d Estimated value of Tajima’s D.

e Simulated value of Tajima’s D given estimates of θ_W_ and θ_π_

f Percent of simulated D values that are lower and higher than the estimated D, respectively.

### DNA extraction, amplification, and sequencing

A ~4-cm^2^ piece of leaf tissue (excluding the midvein) was flash frozen in liquid N_2_, lysed with two 6-mm glass beads at a frequency of 30 Hz for 30 sec, and then genomic DNA was extracted and purified using a QIAGEN DNeasy Plant Mini kit; we used Tris-EDTA (pH 8.0) heated to 65° to elute DNA rather than the elution buffer provided. All DNA samples were quantitated using a Qubit and diluted to a final concentration of 10 μg/mL. We randomly selected seven nuclear genes from an unannotated transcriptome of *I. hederacea* (made possible by The 1KP Project; http://www.onekp.com). Primers were designed using a combination of Primer3Plus (http://www.bioinformatics.nl/cgi-bin/primer3plus/primer3plus.cgi) and NetPrimer (http://www.premierbiosoft.com/netprimer/index.html) to amplify short fragments (~500−800 bp) from within these nuclear genes; although the sequences we use do not represent entire genes, for simplicity, we refer to them as nuclear loci throughout. A list of primer sequences can be found in Supporting Information, Table S1.

All seven loci were amplified in 20-μL reaction volumes (using final concentrations of 1X *Taq* buffer, 2.6 mM MgCl_2_, 0.25 mM dNTPs, 0.25 mM forward and reverse primers, 1U Fermentas *Taq* polymerase [Fisher Scientific], and 20 ng DNA template) on an Eppendorf Mastercycler using the following polymerase chain reaction (PCR) protocol; 2 min at 95°, then 45 sec at 95°, 30 sec at 55° (−0.2° each cycle), 1 min at 72°, for 30 cycles, then 30 sec at 95°, 30 sec at 49°, 1 min at 72°, for 10 cycles, with a final extension of 10 min at 72°. PCR products were verified on 1.5% ethidium bromide stained agarose gels and then purified by adding 2 U of Fermentas SAP and 4 U of Fermentas *Exo*I to the remaining PCR products, and incubating them for 30 min at 37°. Sequencing was carried out in 10-μL reaction volumes (1X BigDye buffer, 0.5 mM primer, 3 μL of purified PCR product, 0.5 μL of BigDye [Applied Biosystems]) using the following protocol; 1 min at 96°, then 10 sec at 96°, 5 sec at 50°, 4 min at 60°, for 45 cycles, then 4 min at 60°. Sequences were obtained using an Applied Biosystems 3730 DNA Analyzer by the Centre for the Analysis of Genome Evolution and Function (University of Toronto).

We sequenced both the forward and reverse strands of DNA loci, then assembled and aligned them using Sequencher v 4.6 (Gene Codes), and edited all chromatographs and verified polymorphic sites (both single-nucleotide polymorphisms [SNPs] and heterozygous sites) manually to ensure robust scoring. Each individual haplotype was reconstructed from unphased diploid sequences using PHASE (Stephens *et al.* 2001), as implemented in the program DnaSP v 5.0 (Librado and Rozas 2009). We determined the open reading frame for coding regions by using a combination of intron locations (identified by aligning the consensus sequence we obtained to the transcriptome sequence), blastx (modified version of BLAST that searches protein databases using translated nucleotide sequences), and GeneMark-E (Borodovsky and McIninch 1993), which predicts open reading frames.

### Population structure

We previously explored population structure using dominant amplified fragment-length polymorphisms (AFLP) markers (Campitelli and Stinchcombe 2013a), but we chose to verify these patterns with nucleotide SNPs to eliminate issues of dominance, homoplasy, and repeatability on conclusions about population structure. We first used the generalized Bayesian clustering method of InStruct (Gao *et al.* 2007), which is based on a similar algorithm to the widely used program STRUCTURE (Pritchard *et al.* 2000) but allows for both admixture and inbreeding within populations, which is suitable for a self-compatible plant that exhibits a range of selfing such as *I. hederacea*. InStruct uses a Markov Chain Monte Carlo algorithm to probabilistically assign individuals to a cluster based on their genetic profiles, and searches for the optimal number of genetic clusters (K) to describe the data. We explored K = 1 to K = 20 clusters and determined the optimal number of clusters based on the deviance information criterion (Gao *et al.* 2007). For each hypothesized K, we used 1,000,000 iterations with a burn-in length of 50,000, and ran three independent chains for each hypothesized K to ensure model convergence. Because SNPs within a locus are expected to be in linkage disequilibrium (LD; see below for an analysis of LD), we randomly selected a single SNP from each locus and reran the entire analysis; we found genetic structure to be almost identical to the full analysis, and hence we only report results for the full set of SNPs.

We examined whether our populations displayed IBD (Wright 1943). We used GenAlEx v 6.5b3 (Peakall and Smouse 2012) to calculate pairwise values of Nei’s genetic distance (Nei’s D), population genetic differentiation *F*_ST_, and the linearized-*F*_ST_ transformation (Lin*F*_ST_ = *F*_ST_/(1 – *F*_ST_); Slatkin 1995) and tested whether they were significantly correlated with pairwise geographic distance (GD) by using GenAlEx. We computed the Pearson product-moment correlation coefficient (*R*_xy_) for the three pairs of matrices (Nei D × GD, *F*_ST_ × GD, and Lin*F*_ST_ × GD), and used Mantel tests consisting of 9999 permutations to generate a null distribution of *R*_xy_ for each pair of matrices, for which the observed *R*_xy_ was then compared to for statistical significance.

### Analysis of polymorphic sites

In all subsequent analyses of nucleotide polymorphism, we estimate parameters of nucleotide diversity for silent sites only (synonymous sites and noncoding regions). We first describe the levels of nucleotide diversity both within and across *I. hederacea* populations in NA. To do this, we calculated pairwise nucleotide differences (θ_π_; Tajima 1989) and the number of segregating sites (θ_w_; Watterson 1975) for each population and assessed significance using coalescent simulations as implemented in the program SITES (Hey and Wakeley 1997). To test whether the observed amount of genetic diversity deviates from neutral expectations, we estimated average Tajima’s D (Tajima 1989), and then compared the estimated value with a simulated mean generated from 10,000 permutations of the dataset as implemented in the program HKA (Hey and Wakeley 1997); locus names are randomly rearranged and Tajima’s D is subsequently calculated with each permutation. We determined a significant value if the estimated Tajima’s D was greater than or less than 95% or 5% of the permuted values, respectively. We additionally determined population-wide estimates of Fu’s *F*_S_ (Fu 1997) and Ramos-Onsins and Rozas’s R_2_ (Ramos-Onsins and Rozas 2002), to cross-check our results for Tajima’s D; following recommendations from Ramos-Onsins and Rozas (2002) and Ramirez-Soriano *et al.* (2008), who found them to be powerful tests of population expansion over a large range of values and significance of these statistics were assessed with the use of 10,000 coalescent simulations in DnaSP.

We next explored whether there were any associations between nucleotide diversity and geographic location of a population or leaf shape morph. To investigate possible geographic patterns in diversity, we assessed the diversity indices (θ_π_ and θ_W_) in two ways: First, we regressed the within-population means of diversity against geographic location using both latitude and longitude as independent variables. Second, we split the populations into *north* and *south* groups (12 *north* and 12 *south* populations) depending on their geographic location with respect to the clinal boundary (determined in Campitelli and Stinchcombe 2013a), and statistically compared nucleotide diversity between regions using a Kruskal-Wallis test (Proc Nonpar1way; SAS Institute, Cary, NC). To explore the level of genetic diversity within each leaf morph, we split all of the individuals into groups corresponding to their leaf shape genotype, and similarly compared levels of diversity using a Kruskal-Wallis test.

### Genetic differentiation and LD

To examine how nucleotide variation is partitioned in NA populations of *I. hederacea*, we used an analysis of molecular variation as implemented in GenAlEx 6.5 (Peakall and Smouse 2012). We further estimated both population genetic differentiation and the inbreeding coefficient (*F*_ST_ and *F*_IS_, respectively; Weir and Cockerham 1984) using GenAlEx. To determine statistical significance, we tested *F*_ST_ and *F*_IS_ values against a null distribution generated from 9,999 permutations using GenAlEx. We used our estimate of *F*_IS_ to calculate the average *SR* with the following equation,

$$SR=\frac{2F_{\text{IS}}}{1+F_{\text{IS}}}\times 100$$,

as outlined by Hartl and Clarke (1989). As a comparison, we also include the population-wide *SR* obtained from Instruct.

We estimated individual *F*_ST_ values for each of the polymorphic sites across all sequence data by using Arlequin v 3.5 (Excoffier and Lischer 2010) to obtain a distribution of *F*_ST_ values to use as a point of comparison for the single-locus leaf shape polymorphism. We tested for significance of each *F*_ST_ value against a null distribution generated from 10,000 permutations in Arlequin. Because we expected some level of LD between SNPs (especially within a sequenced fragment), we calculated pairwise LD (*r*^2^) between all SNPs using the ‘genetics’ (Warnes 2013) and ‘LDheatmap’ (Shin *et al.* 2006) packages offered in R (R Core Team 2013). Given the potential for significant LD between loci to lead to nonindependent *F*_ST_ estimates, we then removed all SNPs showing any significant LD from our dataset and recalculated the *F*_ST_ distribution of remaining SNPs to explore whether this distribution was different. Finally, we estimated *F*_ST_ at the leaf shape locus with GenAlEx, using only the samples included in the present study, to compare the strength of differentiation between the leaf shape locus and DNA sequence data.

## Results

### Population structure

**Figure 2:**
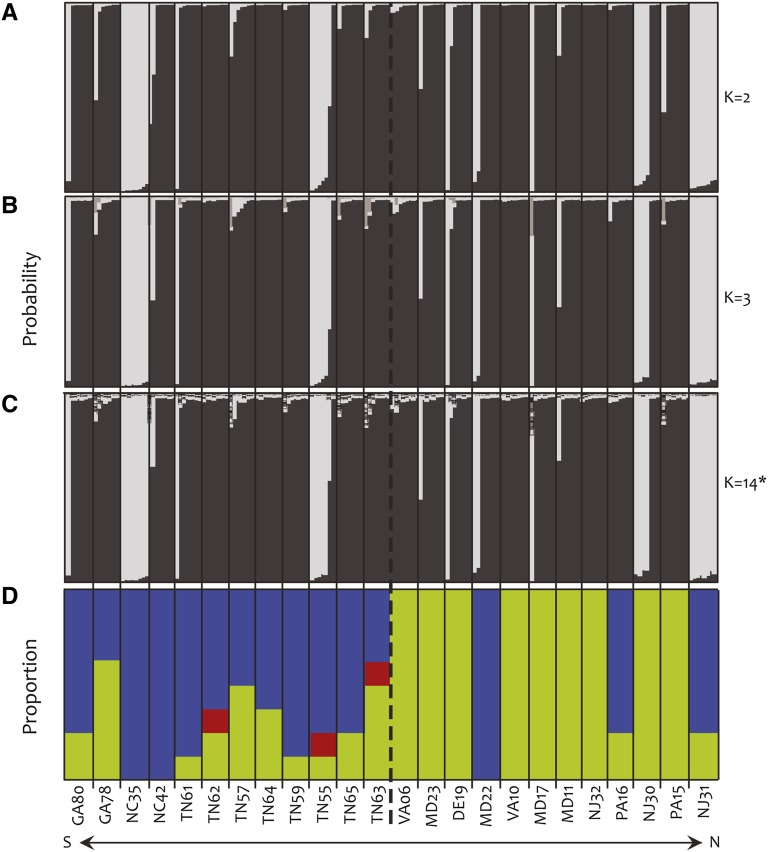
(A–C) Posterior probabilities from InStruct for *I. hederacea* from 24 populations in North America, organized by latitude of origin. The number of hypothesized genetic clusters (K) is given for each. (D) The proportion of each leaf shape genotype within a given population. Each stacked bar in (A), (B), and (C) represents a single individual and the size of a stack represents the fraction of its genome assigned to a given cluster (clusters appear in different shades of gray). There appear to be two main clusters, although K = 14 was identified as the optimal number of clusters. In (D), the blue portion represents entire-shaped individuals, green portions represent lobed individuals, and red portions represent heterozygotes. The dashed line represents the boundary separating northern from southern populations based on a cline analysis done in Campitelli and Stinchcombe 2013a.

We used a Bayesian clustering method to explore the population genetic structure of *I. hederacea* in its NA range. Of the K = 20 clusters we tested, InStruct revealed two major genetic clusters that are patchily distributed by both latitude (Figure 2A) and longitude (not shown). InStruct identified K = 14 to be the optimal number of clusters; however, the addition of K = 3 through 14 failed to resolve the two major clusters much further, and appeared to only explain minor differences between individuals (Figure 2, B and C). A principal coordinates analysis confirmed the main results from InStruct, and so we describe and present them in the supplemental material.

Clustering did not correspond to geographic location (Figure 2); individuals assigned to both major genetic clusters are found in the north and the south. Furthermore, genetic clustering did not correspond to the proportion of each leaf shape in populations (compare Figure 2, A−C and D). For example, populations NC35 and NC42 are fixed for heart-shaped individuals but have individuals assigned to opposite genetic clusters. Furthermore, populations MD22 and VA10 are fixed for opposite leaf shapes but share a similar genetic clustering. We detected little genomic evidence of outcrossing; 89% of the individuals we sampled display at least 95% assignment to one of the two dominant genetic clusters (*i.e.*, <5% of their genomes are from alternative genetic clusters based on the seven loci we sampled).

**Figure 3:**
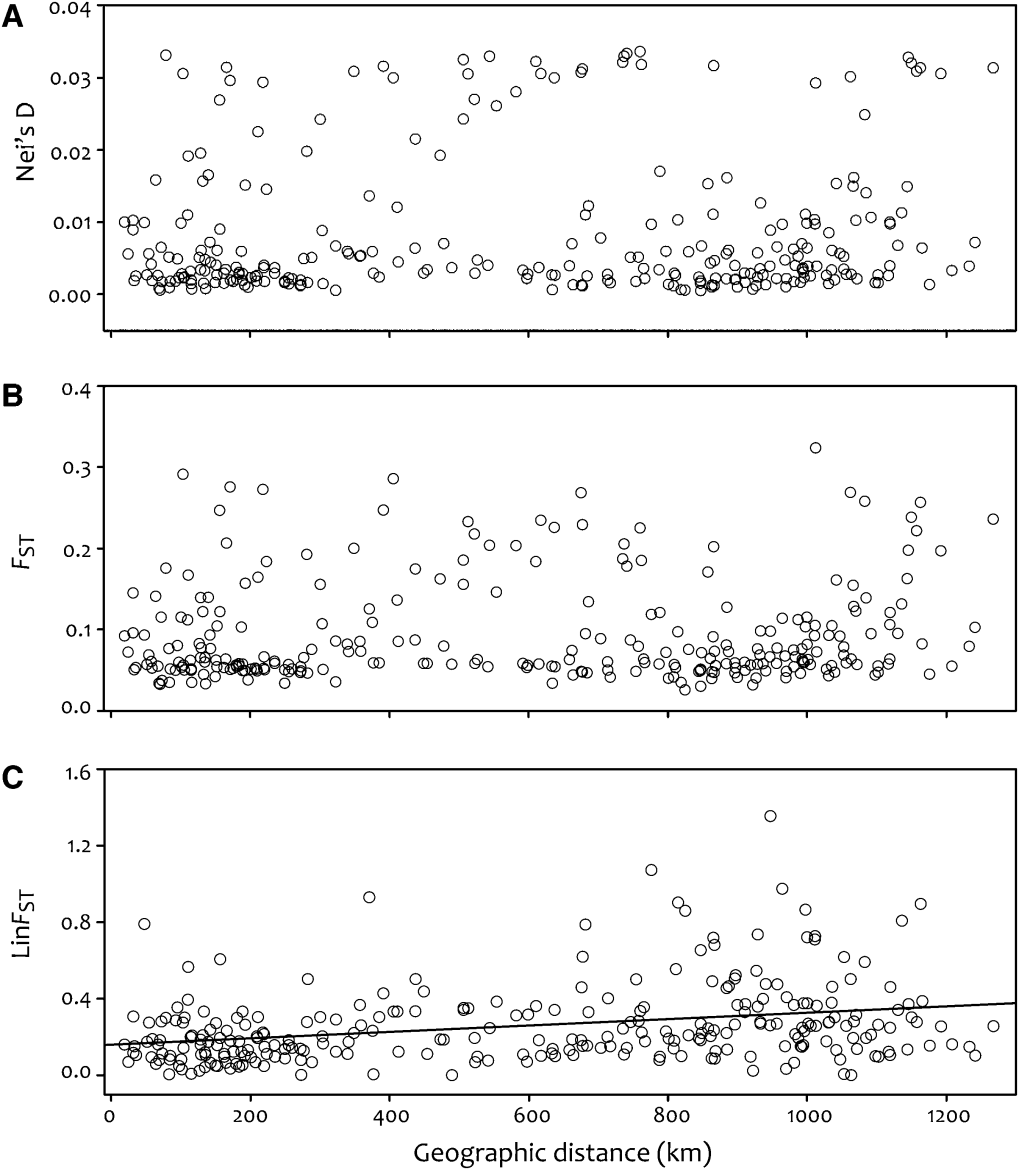
Isolation-by-distance estimates plotting pairwise (A) Nei’s genetic distance, (B) *F*_ST_, and (C) linearized-*F*_ST_ against pairwise geographic distance. Marginal isolation-by-distance (IBD) was found for Nei’s D (*R*_xy_ = 0.072, *P* = 0.10) and *F*_ST_ (*R*_xy_ = 0.074; *P* = 0.088), and significant IBD was detected using linearized-*F*_ST_ (*R*_xy_ = 0.30, *P* = 0.001).

Depending on the parameter considered, we found mixed results for IBD; IBD was marginally significant for Nei’s D (*R*_xy_ = 0.072, *P* = 0.10; Figure 3A) and *F*_ST_ (*R*_xy_ = 0.074; *P* = 0.088; Figure 3B) and significant for Lin*F*_ST_ (*R*_xy_ = 0.30, *P* = 0.001; Figure 3C).

### Patterns of nucleotide diversity

**Figure 4:**
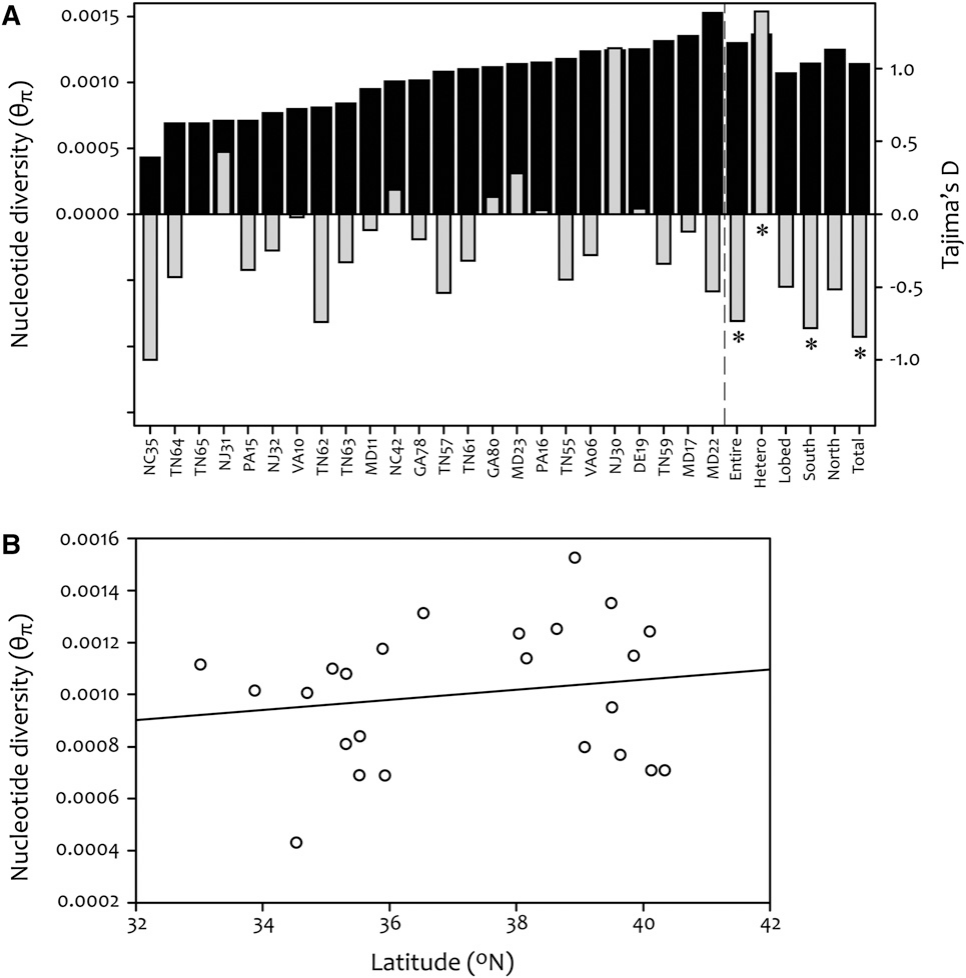
(A) Pairwise nucleotide diversity (dark gray bars) and Tajima’s D (light gray bars) for each population, each leaf shape genotype, each region (based on the clinal boundary determined in Campitelli and Stinchcombe 2013a), and the population-wide estimates (total). (B) Regression of nucleotide diversity against latitude (*R*^2^ = 0.03, *F* = 0.65, *P* = 0.43). Tajima’s D estimates with an asterisk indicate significance at the *P* = 0.05 level.

Population wide estimates of silent site nucleotide diversity for *I. hederacea* were low (θ_W_ = 0.00324 and θ_π_ = 0.00114, *i.e.*, an average of 1.14 nucleotide differences per 1000 bp of DNA between two randomly chosen individuals), with individual populations exhibiting a range of diversity levels (Figure 4A and Table 1). When we grouped populations by their position in the leaf shape cline, we find no difference in nucleotide diversity between northern and southern populations (Kruskal-Wallis; χ^2^ = 0.004, *df* = 1, *P* = 0.95; Figure 4A). Furthermore, a regression analysis revealed that there were no significant correlations of nucleotide diversity with latitude (*R*^2^ = 0.03, *F* = 0.65, *P* = 0.43; Figure 4B), or longitude (*R*^2^ = 0.01, *F* = 0.25, *P* = 0.62; not shown); latitude and longitude reveal similar results, likely because they are significantly correlated for our populations (*R*^2^ = 0.65, *F* = 40.6, *P* < 0.0001). Our results show that leaf shape genotype was likewise not a good predictor of nucleotide diversity (Kruskal-Wallis; χ^2^ = 1.09, *df* = 2, *P* = 0.58; Figure 4A). Summary statistics for each of the seven sequenced loci can be found in Table S2. The lack of geographical or leaf shape patterns associated with diversity make it difficult to determine the expanding front or direction of colonization.

We found that Tajima’s D did not deviate from neutrality for any individual population (Figure 4A and Table 1). However, the overall excess of segregating polymorphisms relative to pairwise differences for all populations combined produced a significantly negative multilocus estimate of Tajima’s D (Figure 4A and Table1). Observed values for Fu’s *F*_S_ and Ramos-Onsins and Rozas’s R_2_ are both significantly smaller than their corresponding simulated mean (obs *F*_S_ = −24.05, sim *F*_S_ = -0.99, *P* < <0.001; obs R_2_ = 0.04, sim R_2_ = 0.09, *P* = 0.01), in agreement with our results for Tajima’s D. We detected a significant deviation from neutrality in southern populations, but not in the north (Figure 4A). Because leaf shape genotype is significantly correlated with latitude, we likewise found that heart shaped genotypes deviated from neutrality while lobed genotypes did not (Figure 4A). Heterozygotes displayed a strong positive Tajima’s D (Figure 4A); however, there were only three heterozygote individuals in our sample such that our power to detect rare sites is severely reduced (*i.e.*, all polymorphic sites had a frequency of at least 33%). Four loci showed significantly negative Tajima’s D, and one showed positive Tajimas’ D, while the remaining two did not deviate from neutral expectations (Table S2).

### Genetic differentiation and LD

A global estimate of Wright’s inbreeding coefficient revealed a high level of inbreeding (*F*_IS_ = 0.86, *P* < 0.0001), yielding an elevated population-wide estimate of selfing (*SR* = 92% when calculated from *F*_IS_, and 93.7% when estimated by Instruct). An analysis of molecular variation of all populations confirmed a lack of admixture; 72% of the molecular variation is partitioned among individuals, 16% partitioned among populations, and the remaining 12% within individuals.

**Figure 5:**
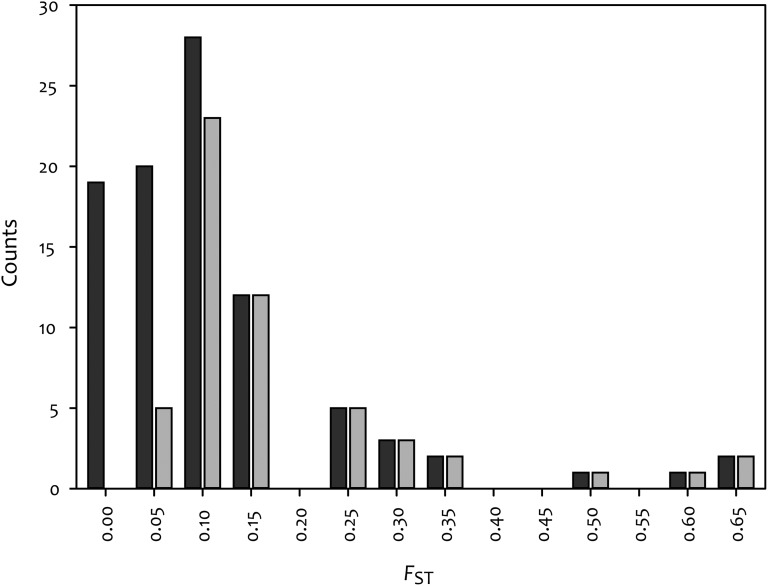
Histogram showing the *F*_ST_ distribution for all single-nucleotide polymorphisms (dark gray bars) and only those that have a significant *F*_ST_ at the *P* = 0.05 level (light gray bars).

A multilocus estimate of global genetic differentiation revealed significant population structure with *F*_ST_ being greater than a null distribution (*F*_ST_ = 0.15, *P* < 0.0001). Individual SNP sites (93 total SNPs), which are directly comparable with the leaf shape locus, exhibit a range of *F*_ST_ values, with about 50% showing significant genetic differentiation (Figure 5). We detected significant LD between 30 of the 93 SNPs in our dataset (Figure S1A). After removing these, we found that the distribution of *F*_ST_ nearly mirrored that for all SNPs (Figure S1B), so we base our interpretations on the whole set. Despite a strong overall signal of global genetic differentiation at SNPs, *F*_ST_ at the leaf shape locus was nearly four times greater (*F*_ST_ = 0.57, *P* < 0.0001) and was in the 96th percentile of the range of *F*_ST_ values for the SNPs. At the leaf shape locus, 40% of the variation was partitioned within populations, 57% among populations and the remaining 3% within individuals.

## Discussion

To understand the historical processes that may have been responsible for patterns of phenotypic and genetic variation in *I. hederacea*, we assessed nucleotide diversity across ~5 kb of DNA sequence for a large representative NA sample. Our analyses revealed three major findings: First, we detected significant population genetic structure, but genetic clusters were patchily distributed and not associated with geography or leaf shape. Second, we found that levels of nucleotide diversity are overall low, and patterns are consistent with population expansion (overall negative value of Tajima’s D indicating the accumulation of rare variants). Third, we found significant genetic differentiation at sequenced loci, but this was not enough to explain the much stronger differentiation at the leaf shape locus. Below we highlight two potential demographic models that are consistent with our sequence data—a founder event associated with colonization of NA, and metapopulation dynamics—and describe the challenge of detecting genetic evidence for each hypothesis. Finally, we address how our current results strengthen our previous interpretation of an adaptive leaf shape cline.

### Disjunct population structure and long-distance dispersal

The patchy genetic structure of *I. hederacea* may potentially indicate long-distance dispersal (LDD). For example, four of the populations we sampled are mostly assigned to their own genetic cluster (NC35, TN55, NJ30, and NJ31; Figure 2A) and are more closely related to one another than they are to the remaining 20 populations (see PCoA in supplemental material; Figure S2). These four populations are all in closer geographic proximity to one of the other 20 populations than they are to one another, suggesting potential LDD events, in agreement with past suggestions of agriculturally mediated dispersal (Epperson and Clegg 1986; Campitelli and Stinchcombe 2013a).

Largely disjunct population structure similar to the type we have observed has been used to infer long-distance dispersal (Gugger *et al.* 2008). For example, Petit *et al.* (1997) proposed that infrequent LDD could explain why haplotypes of two species of *Quercus* (oak) were distributed in clumps in areas of recolonization after the last glacial retreat in Europe, which potentially suggests that *I. hederacea* populations are the result of random colonization events. However, cases of such irregular genetic subdivision are rare (Gugger *et al.* 2008) and easier to justify for long-lived perennials with known dispersal mechanisms. *I. hederacea* does not depend on nonhuman vectors for seed dispersal, and its annual life cycle suggests that other haplotypes can easily invade populations.

Long-distance dispersal is expected to reduce overall IBD by facilitating gene flow and thus eroding the buildup of genetic differentiation. Our results only partially support the notion of reduced IBD under a model of rare LDD events; pairwise Nei’s D and *F*_ST_ show nonsignificant trends of IBD, and linearized-*F*_ST_ displays significant IBD (Figure 3). In addition, we found significant population-wide differentiation (*F*_ST_ = 0.15; Figure 5), signifying genetic differentiation between populations; however, one might expect even higher *F*_ST_ given a high level of selfing. The potential inconsistency between LDD on one hand, and significant genetic differentiation and IBD on the other, may be explained by a relative lack of outcrossing between individuals following seed dispersal. Our analyses suggest that outcrossing between individuals is uncommon, even within populations; our evidence shows that ~90% of individuals we assessed have genomes that are less than 5% recombined. In this sense, each population may consist of multiple “populations” if individuals are rarely outcrossing. Furthermore, sixfold more of genetic variation we detected is partitioned between individuals (72%) compared to within individuals (12%). These results are additionally supported by the high average selfing rates of 92% and 93.7% (depending on how it is estimated) that we found. Our findings are in agreement with previously documented selfing rates using a combination of progeny arrays (93%; Ennos 1981), or *F*-statistics from 173 AFLP loci (74%, reanalysis of data in Campitelli and Stinchcombe 2013a); although Hull-Sanders *et al.* 2005 found slightly lower *SRs* (~63%) using allozyme markers (this discrepancy likely arises from differences in the methods, sample sizes, and markers used to estimate selfing). Combined, these findings suggest a general lack of outcrossing, which may contribute to the discontinuous population structure of *I. hederacea*.

### Low molecular diversity and population expansion

Although self-compatible species typically show a reduction in genetic diversity (*e.g.*, Ness *et al.* 2010; Foxe *et al.* 2010; Gonzales *et al.* 2012), the diversity levels in *I. hederacea* (θ_π_ never exceeding 0.0016, Figure 4A) are remarkably low. For comparison, the close relative *I. purpurea* exhibits over an order of magnitude more silent site nucleotide diversity (Gonzales *et al.* 2012), albeit at loci in the anthocyanin biosynthetic pathway rather than randomly chosen. Additionally, *I. hederacea* ranks in approximately the bottom 25% of a wide range of both plant and animal taxa with respect to genetic diversity; within plants, only long-lived tree species exhibit lower variation (Leffler *et al.* 2012). Our diversity estimates represent perhaps some of lowest recorded for an herbaceous annual plant, even among primarily selfing species (Gonzales *et al.* 2012). Low levels of genetic diversity such as that for *I. hederacea* suggests that historical events—such as a founder event associated with the colonization of NA, or repeated bottlenecks, or genome-wide purifying selection—has severely reduced or is maintaining a low level of genetic diversity in *I. hederacea* in NA. Given that we examined synonymous sites in loci that are presumably randomly distributed throughout the genome, it is unlikely that purifying selection on these sites themselves or physically linked sites would be responsible for maintaining such low levels of diversity across the genome, and so we do not consider this possibility further.

Although segregating site variation was also quite low, we detected significantly more rare variants than pairwise differences compared to a neutral model (Tajima’s D = −0.84, Figure 4). Negative Tajima’s D values are associated with an accumulation of rare alleles, and hence are often interpreted as indicating rapid population expansion. Interestingly, individual populations showed both negative and positive values of Tajima’s D, but none of them were different from a neutral model, suggesting that although we cannot reject neutrality for individual populations, taken together they suggest *I. hederacea* may have recently undergone population expansion in NA.

### Population history of *I. hederacea* in NA

The patterns of nucleotide diversity and population structure that we have observed suggest at least two nonexclusive hypotheses regarding the population history of *I. hederacea*: (1) It recently colonized NA and (2) NA populations are exhibiting metapopulation dynamics. The particularly low level of genetic variation, and lack of geographical patterns in diversity, may be explained by a recent and severe founder event because we would otherwise predict the buildup of neutral molecular variation beyond the observed levels and expect to detect reductions in diversity along the range expanding front (reviewed by Soltis *et al.* 2006). Several authors have suggested that *I. hederacea* is either a released ornamental from the tropics or migrated with the historical trade of corn from Mexico into the United States (Strausbaugh and Core 1964; Long and Lakela 1971; Wunderlin 1982), which is consistent with the founder hypothesis. Severe reductions of diversity often are associated with invasion; for example, genetic diversity is reduced by nearly 35% in NA populations of *I. purpurea* compared with their native Mexican populations (Fang *et al.* 2013). Following the colonization of NA, rare LDD (Campitelli and Stinchcombe 2013a; Figure 2 this study) followed by rapid population expansion could explain the patchy genetic structure, and the overall negative value of Tajima’s D.

A variant on the founder event hypothesis is that population structure in NA *I. hederacea* and the two predominant genetic clusters may have been derived from genetic structure in the ancestral range. Under this model, members of both genetic clusters would have been introduced over wide areas throughout the current range, either at once or serially. In this manner, the lack of geographic structure or strong patterns of *F*_ST_ would not be indicative of widespread gene flow or LDD but rather the same genetic variants being introduced widely and remaining extant across a wide range. If true, this population genetic model would imply even stronger selection on leaf shape and life history traits, as they have diverged latitudinally while the patchy population genetic structure has remained intact. However, it remains unclear what constitutes the ancestral range of *I. hederacea*.

Alternatively, populations of *I. hederacea* in NA may be experiencing repeated bottlenecks that maintain low levels of genetic diversity by persistently eliminating rare alleles. Given that *I. hederacea* primarily inhabits recently disturbed sites such as agricultural fields, it is possible that whole populations may be routinely founded, extirpated, then founded again (from the seed bank or LDD seed from another population), leading to a high-turnover rate. These contemporary forces may be causing *I. hederacea* populations to behave more akin to a single large metapopulation, with high extinction and colonization rates driving the genetic diversity and structure patterns we observe throughout its eastern NA range. Under this scenario, recent founder events associated with colonization are not a necessary requirement of the metapopulation hypothesis, because frequent and strong bottlenecks may reduce and maintain a minimal number of haplotypes.

The founder event and metapopulation dynamics scenarios are not mutually exclusive hypotheses, and further exploration of local extinction and colonization rates are required to disentangle the two possibilities. Furthermore, to conclusively determine whether the low genetic diversity results primarily from a founder event or metapolulation dynamics, greater geographic sampling of the molecular genetic diversity across *I. hederacea’s* range will be necessary. However, *I. hederacea’s* major center of occurrence is in the eastern United States, whereas its most closely related congener *I. nil* is found predominantly in Mexico and parts of South America (http://www.gbif.org). Although some analyses suggest taxonomic/phylogenetic uncertainty between these two species (Miller *et al.* 2004), others find well-resolved support for them as separate species (R. Baucom and J. R. Stinchcombe unpublished data), making it unclear what would need to be sampled.

### Revisiting the leaf shape cline

Using markers that are not susceptible to any of the well-known problems of AFLPs, the findings we have presented here strengthen our interpretation of the leaf shape cline as an example of adaptation (Mueller and Wolfenbarger 1999; Mba and Tohme 2005; Arrigo *et al.* 2009). First, we found nearly fourfold stronger genetic differentiation at the leaf shape locus relative to the average for all sequenced loci, and only 3% of the SNPs (of 93 total SNPs) display an equal or greater *F*_ST_, suggesting the action of divergent selection on the leaf locus. Second, there is discordance between the genetic constitution and the prevailing leaf shape of a population; genetic clustering does not predict leaf shape genotype frequencies for populations (Figure 2). Third, the potential LDD leading to a somewhat random genetic structure is not reflected at the leaf shape locus (Figure 2D). Hence, despite the potential role of historical demography and stochastic population dynamics revealed by our analysis of nucleotide variation, we still find that the leaf shape locus is more differentiated than nucleotide sites from a random selection of nuclear loci, strongly implicating that this locus is in fact under selection. This is a particularly interesting finding in this work, given the evidence for forces—demography and stochasticy—that should remove or mask adaptive signals (see for *e.g.*, Domingues *et al.*2012). Initial investigations of physiological differences between leaf shape genotypes, abiotic and biotic agents that might impose selection on leaf shape, and potential pleiotropically linked traits suggest that the agent of selection on leaf shape is still at large (Campitelli and Stinchcombe 2013b; Campitelli *et al.*, 2013).

Our findings on patterns of neutral polymorphism and structure, combined with previous work on life-history and morphological traits (Klingaman and Oliver 1996; Campitelli and Stinchcombe 2013a; Stock *et al.* 2014), suggests that the evolutionary history of *I. hederacea* in NA can be explained by both demographic processes (*e.g.*, infrequent LDD and colonization) and adaptation (*e.g.*, clines for both leaf shape and flowering time). Future work that investigates geographic patterns of genetic variation in the chloroplast genome of *I. hederacea* (chloroplast DNA is maternally inherited through seed only, Petit *et al.* 1997; Gugger *et al.* 2008), may provide useful insight on the importance of rare LDD of seeds in shaping the contemporary patterns of genetic and phenotypic variation in *I. hederacea*.

## Supplementary Material

Supporting Information
