# Supplementary material for: Population Dynamics and Evolutionary History of the Weedy Vine Ipomoea hederacea in North America
Source: G3 (Bethesda). 2014 Jun 3;4(8):1407–16. doi: 10.1534/g3.114.011700 (PMC4132172; doi:10.1534/g3.114.011700)
Supplement: Supporting Information [file supp_4_8_1407__index.html]

Population Dynamics and Evolutionary History of the Weedy Vine Ipomoea hederacea in North America — Supporting Information 

# Population Dynamics and Evolutionary History of the Weedy Vine *Ipomoea hederacea* in North America

## Supporting Information for Campitelli and Stinchcombe, 2014

**Files in this Data Supplement:**

- Supporting Information - Figures S1-S2, File S1, and Tables S1-S2 (PDF, 1 MB)
- Figure S1 - (a) Heatmap showing pairwise linkage disequilibrium for all polymorphic sites. (b) Histogram showing the *F*ST distribution for all SNPs exhibiting non-significant LD (dark grey bars represent all 63 SNPs within this group, and light grey bars show only those that are significant at the *P* = 0.05 level). (PDF, 873 KB)
- Figure S2 - Principle coordinates analysis using pairwise (a) Nei's genetic distance, (b) *F*ST, and (c) linearized-*F*ST. (PDF, 532 KB)
- File S1 - Supplemental material (PDF, 115 KB)
- Table S1 - Primer sequences for each of the seven sequenced loci. (PDF, 82 KB)
- Table S2 - Diversity statistics for each of the sequenced loci. (PDF, 100 KB)
