# Supplementary material for: Population Dynamics and Evolutionary History of the Weedy Vine Ipomoea hederacea in North America
Source: G3 (Bethesda). 2014 Jun 3;4(8):1407–16. doi: 10.1534/g3.114.011700 (PMC4132172; doi:10.1534/g3.114.011700)
Supplement: Supporting Information [file supp_g3.114.011700_011700SI.pdf]

G3·Genes | Genomes | Genetics

Population dynamics and evolutionary history of the weedy vine *Ipomoea hederacea* in North America

Brandon E. Campitelli *
John. R. Stinchcombe §†

* Department of Integrative Biology, College of Natural Sciences, University of Texas at Austin, Austin, TX, 78712
§ Department of Ecology and Evolutionary Biology, University of Toronto, Toronto, ON, Canada, M5S 3B2
† Centre for the Analysis of Genome Evolution and Function, University of Toronto, Toronto ON, Canada, M5S 3B2

Running title: Evolutionary history of *I. hederacea*

Keywords: population expansion, structure, bottlenecks, metapopulation, leaf shape

Correspondence: Brandon E. Campitelli, Address: 2415 Speedway, Patterson Labs, Austin, TX 78712. Phone: (512) 471-3278. E-mail: brandon.campitelli@gmail.com

DNA sequences from this article have been deposited to the GenBank Data Library under accession numbers KJ875975 – KJ877179

DOI: 10.1534/g3.114.011700

Figure S1

a

b

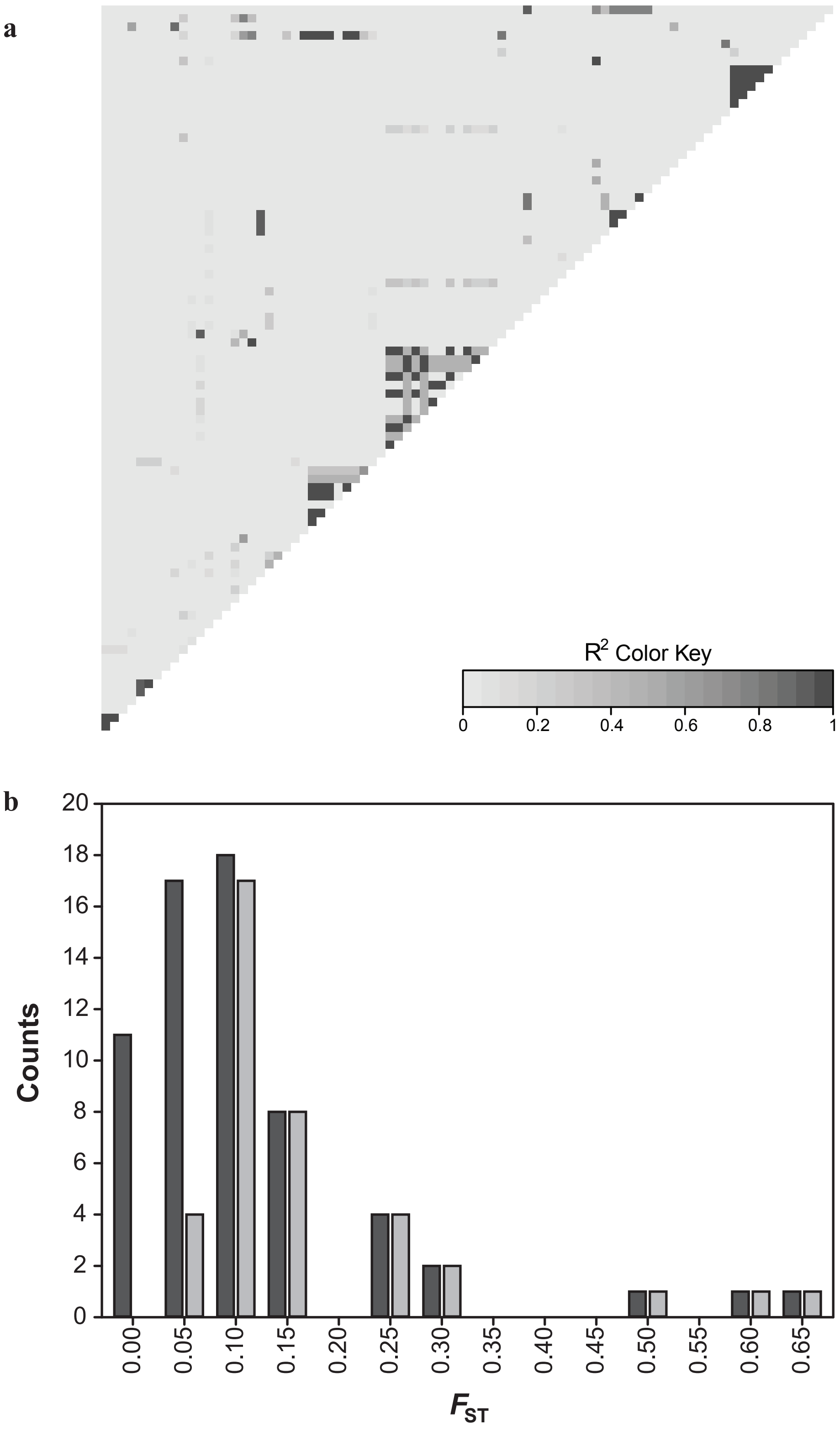

Figure S1. (a) Heatmap showing pairwise linkage disequilibrium for all polymorphic sites. (b) Histogram showing the FST distribution for all SNPs exhibiting non-significant LD (dark grey bars represent all 63 SNPs within this group, and light grey bars show only those that are significant at the P = 0.05 level).

Figure S2

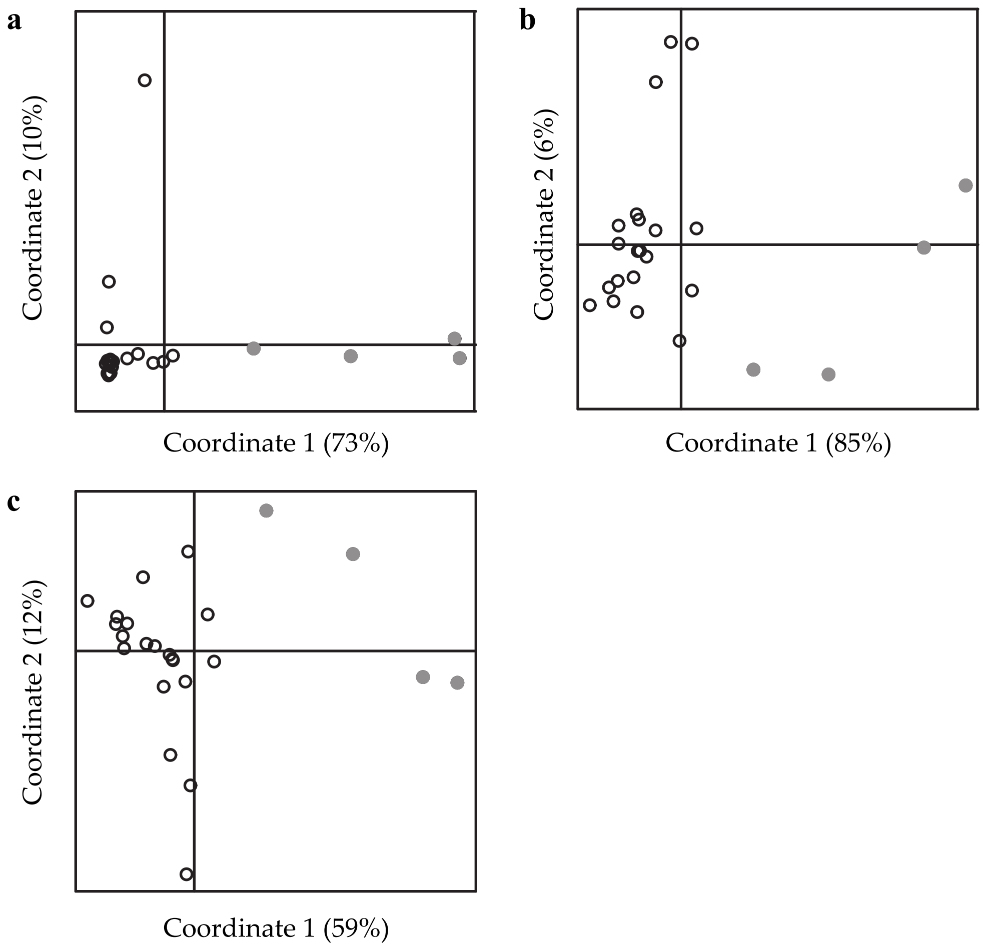

Figure S2. Principle coordinates analysis using pairwise (a) Nei's genetic distance, (b) FST, and (c) linearized-FST. The solid light grey points represent the four populations that contained many individuals assigned to a unique genetic cluster (light grey bars in Fig. 3). The percent of variation explained by each axis is given in parentheses.

File S1

Supplemental material

Principal coordinates analysis

As a supplemental method to explore population structure, we performed a principle coordinates analysis (PCoA), and compared how populations cluster based on various genetic distance parameters to our InStruct analysis above. To perform a PCoA, we used GenAlEx v 6.5b3 (Peackall and Smouse 2012) to first calculate Nei's genetic distance (Nei D), F_{ST}, and the linearized-F_{ST} transformation (LinF_{ST} = F_{ST}/[1-F_{ST}]; Slatkin 1995) between all pairwise combinations of populations. We then performed a PCoA using the covariance-standardized method as implemented in GenAlEx.

Results of PCoA

Our PCoA analyses reinforced our InStruct results by revealing the same dominant genetic groups of populations; the four populations identified using InStruct (NC35, TN55, NJ30 and NJ31; Fig. 2) were separated from all other populations along axis 1 in each PCoA (Fig. S2 a – c). The remaining populations generally clustered closely together, with the exception of a few populations (TN61, GA78 and TN63) showing separation along axis 2; we note that axis 2 explained significantly less than axis 1 in all PCoAs, and these three populations did not show a unique profile in our InStruct analysis.

Table S1 Primer sequences for each of the seven sequenced loci.

Locus	Direction	Sequence (5' to 3')
IH00534	Forward	TGCCATTCTCCCTTTGTTTT
	Reverse	TCCCGGAACTTGTGAAGATT
IH04535	Forward	GCTGTCGGGAACTCAAAGAC
	Reverse	ATCGGTAAATTGTGGGTGGA
IH05255	Forward	CACGAGAGAGAAGGGAGGTG
	Reverse	ACAAATGCCAGCAAGGAATC
IH06033	Forward	GGAACTTTCTTGCCATTTGC
	Reverse	GAAGACACCGATGCAGTGAA
IH06279	Forward	TTCGAGCCGGTCAGATTAAG
	Reverse	GCAAAATCTTGGTTGCCAGT
IH06974	Forward	AAGTCGGGCATTCCACTAGA
	Reverse	TGGGATGTCATCTTTGCTGA
IH16579	Forward	TGGGGCTCTAGTTTCCAGTG
	Reverse	CCAGAAATCCGCCTTTACAA

Table S2 Diversity statistics for each of the sequenced loci.

Locus	Total bp	NS bp	Silent bp	θ_W	θ_π	Taj D	Sim D	% low	% high
IH00534	810	549	261	0.00470	0.00023	**-1.95**	-0.019	0.0	100.0
IH04535	726	484	242	0.00126	0.00393	**2.62**	-0.019	99.2	0.8
IH05255	547	306	241	0.00318	0.00083	**-1.30**	-0.004	0.5	99.5
IH06033	774	516	258	0.00000	0.00000	--	--	--	--
IH06279	656	410	246	0.00685	0.00057	**-2.04**	-0.069	0.0	100.0
IH06974	726	230	496	0.00340	0.00020	**-2.09**	0.001	0.0	100.0
IH16579	703	469	234	0.00327	0.00278	-0.26	-0.018	46.1	53.9

Boldface values indicate significance at the $P = 0.05$ level. Diversity indices are the same as in Table 1. NS refers to non-synonymous sites.
