# Supplementary material for: Population Dynamics and Evolutionary History of the Weedy Vine Ipomoea hederacea in North America
Source: G3 (Bethesda). 2014 Jun 3;4(8):1407–16. doi: 10.1534/g3.114.011700 (PMC4132172; doi:10.1534/g3.114.011700)
Supplement: Supporting Information [file supp_g3.114.011700_FigureS1.pdf]

Figure S1

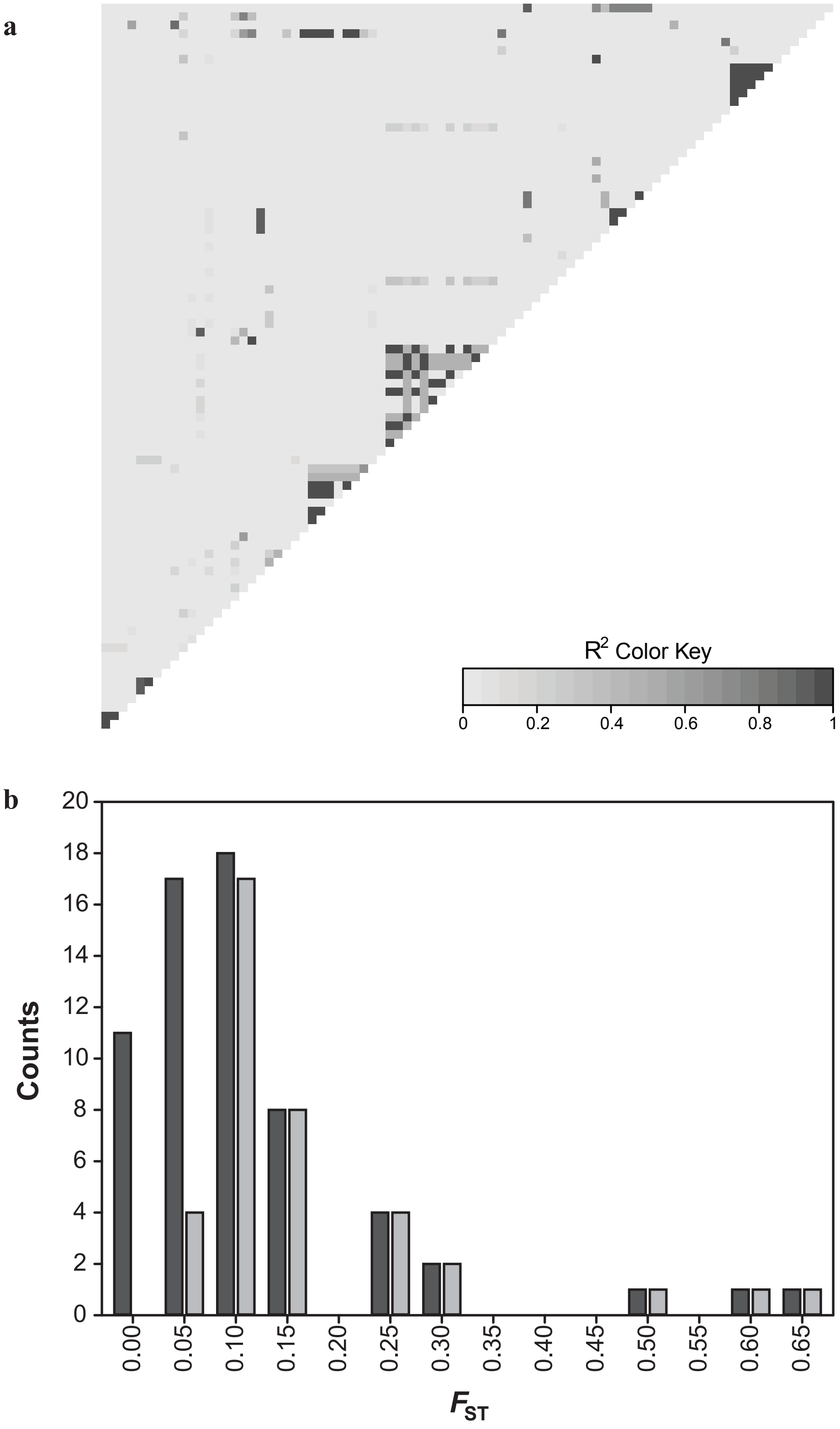

Figure S1. (a) Heatmap showing pairwise linkage disequilibrium for all polymorphic sites. (b) Histogram showing the FST distribution for all SNPs exhibiting non-significant LD (dark grey bars represent all 63 SNPs within this group, and light grey bars show only those that are significant at the P = 0.05 level).
