# Supplementary material for: Population Dynamics and Evolutionary History of the Weedy Vine Ipomoea hederacea in North America
Source: G3 (Bethesda). 2014 Jun 3;4(8):1407–16. doi: 10.1534/g3.114.011700 (PMC4132172; doi:10.1534/g3.114.011700)
Supplement: Supporting Information [file supp_g3.114.011700_FigureS2.pdf]

Figure S2

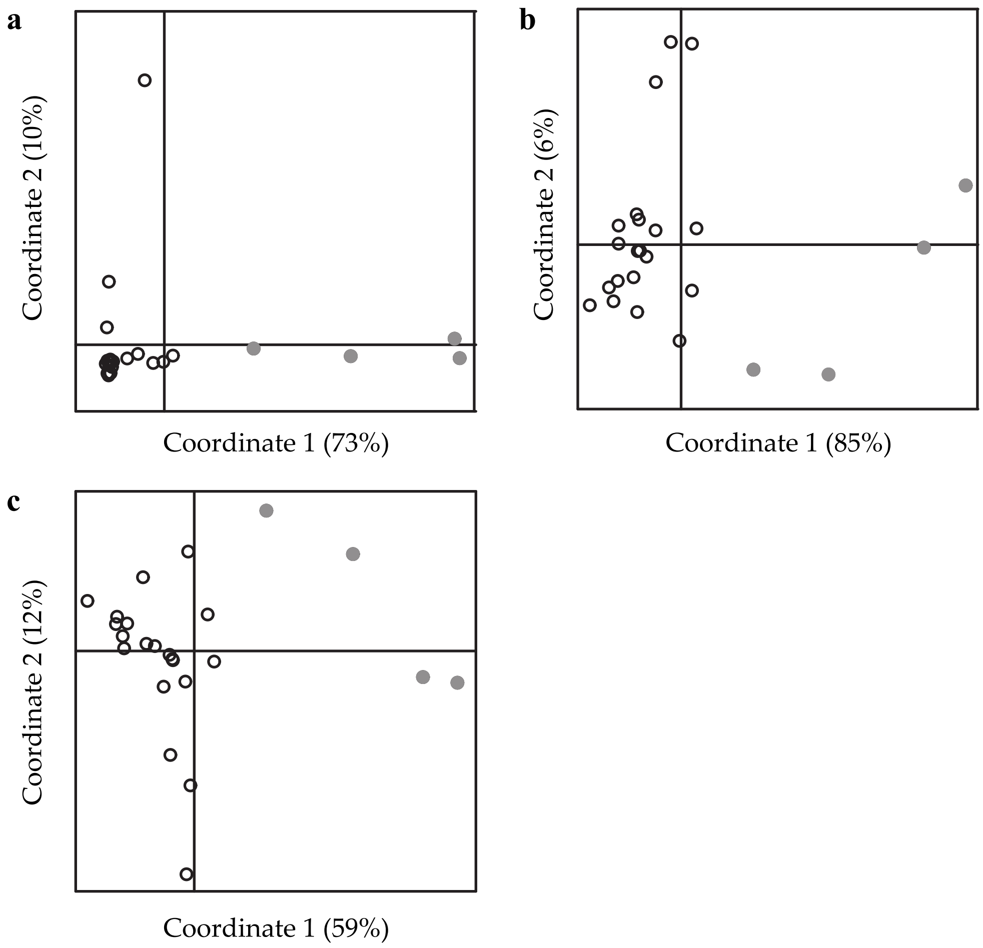

Figure S2. Principle coordinates analysis using pairwise (a) Nei's genetic distance, (b) FST, and (c) linearized-FST. The solid light grey points represent the four populations that contained many individuals assigned to a unique genetic cluster (light grey bars in Fig. 3). The percent of variation explained by each axis is given in parentheses.
