# Supplementary material for: Population Dynamics and Evolutionary History of the Weedy Vine Ipomoea hederacea in North America
Source: G3 (Bethesda). 2014 Jun 3;4(8):1407–16. doi: 10.1534/g3.114.011700 (PMC4132172; doi:10.1534/g3.114.011700)
Supplement: Supporting Information [file supp_g3.114.011700_FileS1.pdf]

File S1

Supplemental material

Principal coordinates analysis

As a supplemental method to explore population structure, we performed a principle coordinates analysis (PCoA), and compared how populations cluster based on various genetic distance parameters to our InStruct analysis above. To perform a PCoA, we used GenAlEx v 6.5b3 (Peackall and Smouse 2012) to first calculate Nei's genetic distance (Nei D), F_{ST}, and the linearized-F_{ST} transformation (LinF_{ST} = F_{ST}/[1-F_{ST}]; Slatkin 1995) between all pairwise combinations of populations. We then performed a PCoA using the covariance-standardized method as implemented in GenAlEx.

Results of PCoA

Our PCoA analyses reinforced our InStruct results by revealing the same dominant genetic groups of populations; the four populations identified using InStruct (NC35, TN55, NJ30 and NJ31; Fig. 2) were separated from all other populations along axis 1 in each PCoA (Fig. S2 a – c). The remaining populations generally clustered closely together, with the exception of a few populations (TN61, GA78 and TN63) showing separation along axis 2; we note that axis 2 explained significantly less than axis 1 in all PCoAs, and these three populations did not show a unique profile in our InStruct analysis.
