# Supplementary material for: Population Dynamics and Evolutionary History of the Weedy Vine Ipomoea hederacea in North America
Source: G3 (Bethesda). 2014 Jun 3;4(8):1407–16. doi: 10.1534/g3.114.011700 (PMC4132172; doi:10.1534/g3.114.011700)
Supplement: Supporting Information [file supp_g3.114.011700_TableS1.pdf]

Table S1 Primer sequences for each of the seven sequenced loci.

Locus	Direction	Sequence (5' to 3')
IH00534	Forward	TGCCATTCTCCCTTTGTTTT
	Reverse	TCCCGGAACTTGTGAAGATT
IH04535	Forward	GCTGTCGGGAACTCAAAGAC
	Reverse	ATCGGTAAATTGTGGGTGGA
IH05255	Forward	CACGAGAGAGAAGGGAGGTG
	Reverse	ACAAATGCCAGCAAGGAATC
IH06033	Forward	GGAACTTTCTTGCCATTTGC
	Reverse	GAAGACACCGATGCAGTGAA
IH06279	Forward	TTCGAGCCGGTCAGATTAAG
	Reverse	GCAAAATCTTGGTTGCCAGT
IH06974	Forward	AAGTCGGGCATTCCACTAGA
	Reverse	TGGGATGTCATCTTTGCTGA
IH16579	Forward	TGGGGCTCTAGTTTCCAGTG
	Reverse	CCAGAAATCCGCCTTTACAA
