# Supplementary material for: Population Dynamics and Evolutionary History of the Weedy Vine Ipomoea hederacea in North America
Source: G3 (Bethesda). 2014 Jun 3;4(8):1407–16. doi: 10.1534/g3.114.011700 (PMC4132172; doi:10.1534/g3.114.011700)
Supplement: Supporting Information [file supp_g3.114.011700_TableS2.pdf]

Table S2 Diversity statistics for each of the sequenced loci.

Locus	Total bp	NS bp	Silent bp	θ_W	θ_π	Taj D	Sim D	% low	% high
IH00534	810	549	261	0.00470	0.00023	**-1.95**	-0.019	0.0	100.0
IH04535	726	484	242	0.00126	0.00393	**2.62**	-0.019	99.2	0.8
IH05255	547	306	241	0.00318	0.00083	**-1.30**	-0.004	0.5	99.5
IH06033	774	516	258	0.00000	0.00000	--	--	--	--
IH06279	656	410	246	0.00685	0.00057	**-2.04**	-0.069	0.0	100.0
IH06974	726	230	496	0.00340	0.00020	**-2.09**	0.001	0.0	100.0
IH16579	703	469	234	0.00327	0.00278	-0.26	-0.018	46.1	53.9

Boldface values indicate significance at the $P = 0.05$ level. Diversity indices are the same as in Table 1. NS refers to non-synonymous sites.
